# Prognostic Significance of *KIT* Mutations in Core-Binding Factor Acute Myeloid Leukemia: A Systematic Review and Meta-Analysis

**DOI:** 10.1371/journal.pone.0146614

**Published:** 2016-01-15

**Authors:** Wenlan Chen, Hui Xie, Hongxiang Wang, Li Chen, Yi Sun, Zhichao Chen, Qiubai Li

**Affiliations:** 1 Institute of Hematology, Union Hospital, Tongji Medical College, Huazhong University of Science and Technology, Wuhan, 430022, China; 2 Department of Hematology, Wuhan Central Hospital, Wuhan, 430000, China; 3 Department of Social Medicine, School of Public Health, Tongji Medical College, Huazhong University of Science and Technology, Wuhan, 430030, China; Queen's University Belfast, UNITED KINGDOM

## Abstract

The prognostic significance of *KIT* mutations in core-binding factor acute myeloid leukemia (CBF-AML), including inv(16) and t(8;21) AML, is uncertain. We performed a systematic review and meta-analysis of the effect of *KIT* mutations on the complete remission (CR) and relapse rates and overall survival (OS) of CBF-AML. PubMed, Embase, Web of Science, and the Cochrane Library were searched and relevant studies were included. Negative effect was indicated on relapse risk of CBF-AML (RR [relative risk], 1.43; 95%CI [confidence interval], 1.20–1.70) and t(8;21) AML (RR, 1.70; 95% CI, 1.31–2.21), not on OS of CBF-AML (RR, 1.09; 95% CI, 0.97–1.23), CR (OR [odds ratio], 0.95; 95% CI, 0.52–1.74), relapse risk (RR, 1.12; 95% CI, 0.90–1.41) or OS (RR, 1.03; 95% CI, 0.90–1.18) of inv(16) AML. Subgroup analysis of t(8,21) AML showed negative effect of *KIT* mutations on CR (OR, 2.03; 95%CI: 1.02–4.05), relapse risk (RR, 1.89; 95%CI: 1.51–2.37) and OS (RR, 2.26; 95%CI: 1.35–3,78) of non-Caucasians, not on CR (OR, 0.61; 95%CI: 0.19–1.95) or OS (RR, 1.12; 95%CI: 0.90–1.40) of Caucasians. This study indicates *KIT* mutations in CBF-AML to be included in the initial routine diagnostic workup and stratification system of t(8,21) AML. Prospective large-scale clinical trials are warranted to evaluate these findings.

## Introduction

Acute myeloid leukemia (AML) with recurrent t(8;21)(q22;q22) [abbreviated as t(8;21)] and inv(16)(p13q22)/t(16;16)(p13;q22) [abbreviated as inv(16)] genetic abnormalities are termed as core-binding factor (CBF)-AML. To date, patients with CBF-AML are generally recognized as a favorable cytogenetic AML sub-group [1]. However, approximately 50% of patients with CBF-AML remain incurable, and markers are required to refine the risk stratification of patients at diagnosis and to optimize their treatment [2]. *KIT* mutations, as potential molecular markers, are found in 12–46% of t(8;21) patients and 9–53% of inv(16) patients [3–8]. Observational studies have assessed the impact of *KIT* mutations on the prognosis of t(8;21) and inv(16) AML [3, 5–8]; however, data concerning the prognostic significance of *KIT* mutations have been conflicting thus far. Some studies have shown that the *KIT* mutation is significantly associated with decreased remission duration and overall survival (OS) in CBF-AML patients [4, 5, 8–12], whereas other studies have shown that *KIT* mutations have no obvious effect on CBF-AML clinical outcomes as a group or in subgroups [7, 13–17]. Although the current data do not support the use of *KIT* mutational status in clinical guidance (in terms of therapeutic interventions), the data have been included in the National Comprehensive Cancer Network Guidelines as a prognostic marker, where the *KIT* mutation can transform CBF-AML patients from favorable-risk AML to intermediate-risk AML [18]. In contrast, the International European Leukemia Net currently does not recommend testing *KIT* mutational status as part of an initial routine diagnostic workup [19, 20]. This inconsistency is based on the current prognostic data of *KIT* mutations in CBF-AML patients. Thus, we performed a systematic review and meta-analysis of published studies to investigate the prognostic significance of *KIT* mutations in CBF-AML patients.

## Methods

### Data sources and search strategy

The first direct evidence of *KIT* mutations leading to the development of human acute leukemia was reported in 1998 [21]; therefore, we searched the PubMed, Embase, Web of Science, and Cochrane Library databases for articles published from January 1, 2000 to December 31, 2014. The following keywords were used for the PubMed search: [“Proto-Oncogene Proteins *KIT*” (MeSH) OR free text search terms “*KIT*” (“kit” OR “CD117” OR “Stem Cell Factor Receptor” OR “SCF Receptor” OR “SCFR”)] AND [“Leukemia, Myeloid, Acute” (MeSH) OR free text search terms “acute myeloid leukemia” (“acute nonlymphocytic leukemia” OR “acute nonlymphocytic leukemias” OR “acute myeloid leukemias” OR “acute myeloid leukemia” OR “AML” OR “ANLL”)]. Similar search terms were used for the Embase, Web of Science, and Cochrane Library databases. The reference lists of identified articles were manually searched for additional studies of interest.

### Study selection

The following inclusion criteria were used: published as an original article in English; focused on patients with CBF-AML, included inv(16), t(8.21), or both; and evaluated any prognostic outcome, including OS, relapse rate, or both, according to *KIT* mutational status. The following exclusion criteria were used: published as an editorial, letter, review, expert opinion, or case report; had no available prognostic data; was a subset of articles by the same author (for multiple reports of a single study, only the most recent or most complete article was considered and examined).

Two reviewers (W.L.C and H.X) independently evaluated the titles and abstracts of the identified publications. Potentially relevant articles were retrieved in full. The final inclusion of articles into our systematic review was based on agreement between both reviewers.

### Data extraction and quality assessment

Data on the characteristics of the selected studies were extracted, according to the guidelines presented in “Systematic Reviews of Genetic Association Studies” by Sagoo *et al*. [22]. Between the two investigators, there was > 98% agreement for data extraction. We extracted the first author’s last name, publication date, country in which the study was performed, study population, study period, median patient age and white blood cell (WBC) counts at diagnosis, sex, incidence of *KIT* mutations, study design, outcome data, including hematological complete remission (CR) rate, relapse rate, and OS using the *KIT* mutational status from each study. To minimize publication bias towards articles that only described significant or relevant data, we contacted all authors for additional data on all evaluated CBF-AML prognostic factors.

Two reviewers (W.L.C and F.C.K) independently assessed the study quality. Disagreements were resolved through a joint reevaluation of the original article. The Newcastle-Ottawa Scale (NOS) [23] was used to score the quality of each cohort study.

### Data synthesis and statistical analysis

Meta-analysis was performed with RevMan version 5.2, according to the Cochrane Collaboration recommendations (http://www.cochrane.org/) and with STATA version 12.0 (Stata-Corp, College Station, Texas, USA). We calculated the CR, the relapse rates and 5-year OS for each sub-category within the study. The CR, relapse and OS were defined as described previously [24]. Event-free survival (EFS) was defined as the time from study entry until death, induction failure, or relapse. Disease-free survival (DFS) was defined as the time from induction CR until relapse or death. The odds ratio (OR) was used to determine the probability of CR following induction therapy based on the *KIT* mutational status. The risk ratio (RR) was used to determine the probability of relapse rate and 5-year OS based on the *KIT* mutational status. For relapse risk, we calculated and analyzed the relapse rate prognostic data, including the relapse rate, relapse incidence, cumulative incidence of relapse of the selected studies. An OR or RR > 1 indicated that the *KIT* mutations were significantly associated with worse outcomes. Given the small number of studies, inter-study heterogeneity, or insufficient reporting, we did not pool the results for other outcomes.

Fixed-effects model analysis was first performed for all data, and the difference was considered to be statistically significant when *P* < 0.05, which was also quantitatively assessed using the *I*^*2*^ value for heterogeneity between the studies. *I*^*2*^ = 25%, 50%, and 75% represent mild, moderate, and severe heterogeneity, respectively [25]. If *I*^*2*^ ≤ 50% and indicated that the studies were homogeneous or slightly heterogeneous, the fixed-effects model was used to combine the effect size. Otherwise, the random-effects model was used [26, 27].

Begg’s and Egger’s tests were used to evaluate publication bias in terms of our primary end points: *P* < 0.05 indicated publication bias. We also used the Duval and Tweedie nonparametric trim and fill procedure to further assess the potential effects of publication bias [28].

To assess the impact of study quality, we considered studies to be high quality and calculated separate random-effects pooled RRs if they had estimates of 2-year OS (n = 3) [11, 12, 29] or 4-year OS (n = 1) [4]. Then, we evaluated the influence of each study on the overall estimate by calculating random-effects pooled RRs, omitting one estimate at a time.

## Results

### Literature search

**Fig 1:**
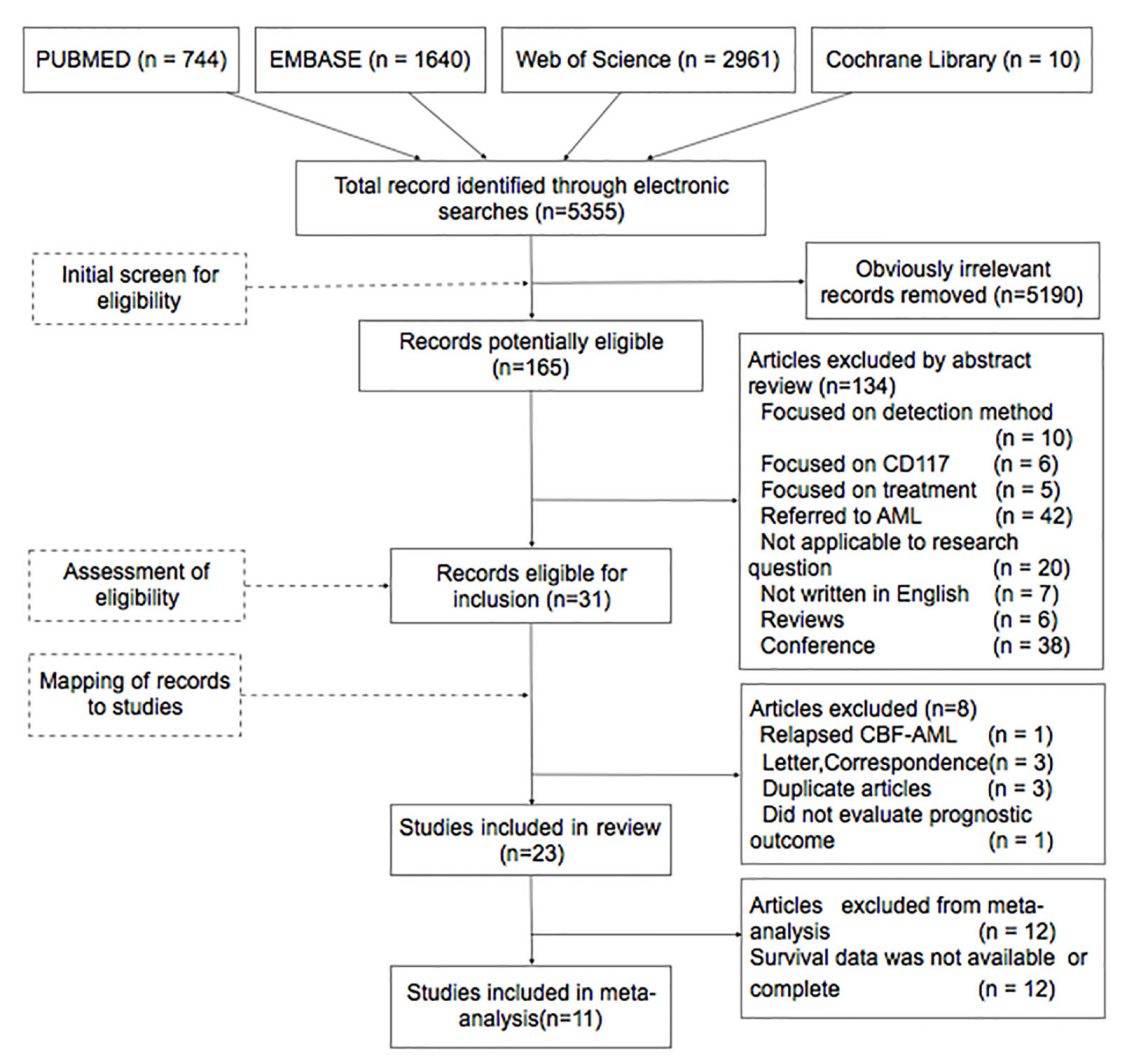
Study flow chart.

After screening titles and reviewing abstracts, we identified 31 potentially relevant articles that focused on *KIT* mutations and CBF-AML (Fig 1). To avoid overlooking AML studies that also described CBF-AML, we rechecked the 42 AML studies that had been excluded following the abstract review, and we found no useful data. Of the 31 candidate articles, we excluded two letters to the editor [30, 31], one correspondence [32], one article that focused on relapsed CBF-AML [33], one that did not evaluate prognostic outcomes [34], and three duplicate articles [35–37]. The remaining 23 articles [3–17, 29, 38–44] were included in the systematic review. Of these, we excluded 12 articles [9, 11–16, 38, 40, 41, 43, 44] from the meta-analysis because they did not provide available survival data. However, we selected two of the excluded studies for sensitivity analysis because they provided 2-year OS data [11, 12]. Citations in the 11 included articles [3–8, 10, 17, 29, 39, 42] and reviews that were associated with CBF-AML were also examined. However, none were included.

### Characteristics of the included studies

The included studies were shown in Table 1. The sample size ranged from 23–354 patients [5, 42]. The 11 included studies included 1 867 patients with CBF-AML [801, inv(16); 1 066, t(8,21)] [3–8, 10, 17, 29, 39, 42]. There were three European studies [3, 5, 6] and six from Asia [4, 8, 10, 17, 29, 42] and two from the United States [7, 39]. There were three retrospective cohort studies [7, 29, 42], and eight prospective cohort studies [3–6, 8, 10, 17, 39], and the frequency of *KIT* mutations in CBF-AML varied from 16–46% [8, 16], with 12–46% [3, 8] and 9–53% [5, 9] in the t(8,21) and inv(16) AML subgroups, respectively. There were two pediatric studies [4, 7], and the others focused on adults.

**Table 1:** Characteristics of studies included in the meta-analysis.

Authors	Median Follow- up	Category	No. ALL of subjects	Male/Female	Age (years)	WBC counting (10^9^/L)	No. *KIT* status		Design of Study^$^
Country									
Publication year	(years)						Wild	Mutated	
Qin *et al*,	0.83	CBF AML	351	NR^&^	NR	NR	223	128	R
China	0.25–7.75	t(8,21)	253	NR	NR	NR	154	99	
2014		inv(16)	98	NR	NR	NR	69	29	
Cairoli *et al*,	4.16	CBF AML	58	48/18	42 (15–60)	24.7 (1.8–277)	43	15	P
Italy	NR	t(8,21)	None^#^	None	None	None	None	None	
2013		inv(16)	58	48/18	42 (15–60)	24.7 (1.8–277)	43	15	
Riera *et al*,	3.7	CBF AML	23	11/12	42.7 (19–64)	NR	16	7	R
Turin, Italy	0.95–11.1	t(8,21)	9	NR	NR	NR	6	3	
2013		inv(16)	14	NR	NR	NR	10	4	
Allen *et al*,	8.3	CBF AML	354	198/156	NR(adult)^^^	NR	254	100	P
UK	1.7–22.1	t(8,21)	199	NR	NR	NR	153	46	
2013		inv(16)	155	NR	NR	NR	101	54	
Paschka *et al*,	6.04	CBF AML	176	94/84	41(18–74)	38.8(1.1–294.9)	110	65	P
Germany	5.3–6.5	t(8,21)	None	None	None	None	None	None	
2013		inv(16)	176	94/84	41(18–74)	38.8(1.1–294.9)	110	65	
Park *et al*,	NR	CBF AML	116	NR	NR(adult)	NR	73	43	P
Korea		t(8,21)	78	NR	NR(adult)	NR	48	30	
2011		inv(16)	38	NR	NR(adult)	NR	25	13	
Pollard *et al*,	5.5	CBF AML	203	106/97	11.8	28.8	165	38	R
America	0.2–9,1	t(8,21)	113	NR	NR	NR	71	19	
2010		inv(16)	90	NR	NR	NR	94	19	
Boissel *et al*,	4.4	CBF AML	103	61/42	33(1–75)	19.1(2–257)	80	16	P
French	NR	t(8,21)	56	NR	NR	NR	44	6	
2006		inv(16)	47	NR	NR	NR	36	10	
Cairoli *et al*,	2.8	CBF AML	67	46/21	NR	NR	36	31	P
Italy	0.8–9.3	t(8,21)	42	2814	40.5(16–76)	8.4(2.1–165)	23	19	
2006		inv(16)	25	42203	51(17–88)	14.6(7.6–277)	13	12	
Paschka *et al*,	5.3	CBF AML	110	35/26	NR(adult)	NR	81	29	P
America	1.6–13.7	t(8,21)	49	24/25	NR(adult)	NR	38	11	
2006		inv(16)	61	35/26	NR(adult)	NR	43	18	
Shimada *et al*,	NR	CBF AML	46	NR	7.5 years(2–15)	14.4(1.65–107.7)	38	8	P
Japanese	NR	t(8,21)	46	NR	7.5 years(2–15)	14.4(1.65–107.7)	38	8	
2006		inv(16)	None	None	None	None	None	None	

^**$**^ P, prospective study

R, retrospective study.

^#^ None, unavailable.

^&^ NR, not reported.

^^^ Age range was 15–59 years

n = 335; > 60, n = 19.

In the included studies, the *KIT* mutations were detected using PCR and direct sequencing; all patients were treated with induction chemotherapy, followed by various consolidation chemotherapies (S1 Table). For patients with age > 60 years, the regimens of normal [5,8,42] or decreased [10] dose were administered. Allogenic hematopoietic stem cell transplantation (Allo-SCT) was used in pediatric patients in CR ≥ 1 [3, 4, 7] and adult patients in CR > 1 [5–8, 10, 17, 29, 39, 42], if a suitable donor was available.

### Quality assessment

Based on the methodology and reported data, 100% of the studies were high quality (NOS score ≥ 6); the average NOS score was 7.3 (S2 Table).

### Outcomes and heterogeneity of the meta-analysis

We analyzed three primary end points (CR, OS, relapse risk) to investigate the prognostic impact of *KIT* mutations on CBF-AML (Table 2). For CR, we evaluated 11 studies [3–8, 10, 17, 29, 39, 42] (1 367 patients): the pooled OR was 1.18 (95% confidence interval [CI]: 0.78–1.78, *P* = 0.31, *I*^*2*^ = 15%). For OS, we evaluated five studies [5–7, 17, 42] (810 patients): the pooled RR was 1.09 (95% CI: 0.97–1.23; *P* = 0.47, *I*^*2*^ = 0%). For relapse risk, nine studies [4, 5, 7, 8, 10, 17, 29, 39, 42] provided relapse rate data, two provided 2-year relapse data [8, 29], one provided 4-year relapse data [4], and six provided relapse data of not less than five years [5, 7, 10, 17, 39, 42]. Therefore, we separately pooled the RRs for 2- and 5-year relapse risk, which were 1.52 (95%CI: 1.31–1.76; *P* = 0.28, *I*^*2*^ = 18%) and 1.43 (95%CI: 1.20–1.70; *P* = 0.50, *I*^*2*^ = 0%), respectively. These results showed that the *KIT* mutations did not impact the CR or OS in CBF-AML cases treated together as an group. However, an analysis of the 2- and 5-year data showed that patients with *KIT* mutations had a significantly higher relapse risk. The *P*-values and *I*^*2*^ statistics for the above data indicated low heterogeneity, and the Begg’s and Egger’s tests indicated no publication bias. CBF-AML patient outcomes, according to the *KIT* mutations, are shown in S3 Table.

**Table 2:** Meta-analysis results.

Outcome	AML	N*	Patients, n	Mut^~^, %	Fixed ^$^	Random ^$^	Heterogeneity		Subgroup differences				Publication bias	
									Fixed ^$^		Random ^$^		Begg	Egger
					RRs 95%CI^#^	RRs 95% CI	*P*	*I^2^*	*P*	*I^2^*	*P*	*I^2^*	*P*	*P*
**CR**														
	CBF AML	11	1367	402(29)	1.18 [0.78, 1.78]	1.19 [0.72, 1.96]	0.31	14%					0.474	0.375
	t(8,21)	9^&^	726	197(27)	1.42 [0.81, 2.49]	1.54 [0.84, 2.86]	0.48	0%					0.386	0.330
	inv(16)	9^^^	654	206(31)	0.95 [0.52, 1.74]	1.03 [0.55, 1.95]	0.48	0%					0.548	0.926
	Total	11	1380		1.17 [0.78, 1.77]	1.27 [0.82, 1.98]	0.54	0%	0.34	0%	0.37	0%		
**OS**														
	CBF AML	5	810	222(27)	1.09 [0.97, 1.23]	1.06 [0.94, 1.19]	0.47	0%					1.000	0.527
	t(8,21)	6	498	113(22)	1.35 [1.09, 1.66]	1.42 [0.95, 2.12]	0.04	58%					0.452	0.023
	inv(16)	8	637	198(31)	1.03 [0.90, 1.18]	1.01 [0.86, 1.18]	0.23	25%					0.902	0.904
	Total	8	1135		1.13 [1.01, 1.27]	1.09 [0.93, 1.27]	0.05	41%	0.03	78%	0.12	58%		
**Relapse Risk**														
2 y	CBF AML	9	982	278(28)	1.52 [1.31, 1.76]	1.54 [1.30, 1.83]	0.28	18%					0.602	0.342
	t(8,21)	8	619	171(28)	1.76 [1.45, 2.12]	1.74 [1.45, 2.10]	0.52	0%					0.108	0.061
5 y	CBF AML	6	781	208(27)	1.43 [1.20, 1.70]	1.46 [1.23, 1.73]	0.5	0%					0.707	0,621
	t(8,21)	5	418	101(24)	1.70 [1.31, 2.21]	1.76 [1.36, 2.26]	0.78	0%					1.000	0.795
	inv(16)	6	387	111(29)	1.12 [0.90, 1.41]	1.18 [0.79, 1.76]	0.07	52%					1.000	0.324
	Total	6	805		1.34 [1.13, 1.59]	1.44 [1.14, 1.84]	0.09	38%	0.02	82%	0.10	63%		

^#^ ORs for CR and RRs for OS and relapse rate.

^&^ In a study by Shimada *et al*., the CR rate is 100% in the patients with or without *c-KIT* mutations.

^^^ In studies by Riera *et al*. and Cairoli *et al*. (2006), the CR rate is 100%.

^$^ Abbreviations for the fixed-effects and random-effects models.

^*^ N: Studies included.

^~^ Numbers of patients with *KIT-*mutations.

### Subgroup analysis of CBF-AML

Though CBF-AML is often considered together in one prognostic group, it has been reported that t(8,21) and inv(16) AML appear to be distinct clinical entities [2, 45]. Accordingly, we analyzed the clinical outcomes (CR, OS and relapse risk) separately by subtype.

For the subgroup analysis of CR, we included nine t(8,21) AML studies [3–5, 7, 8, 10, 29, 39, 42] (726 patients) and nine inv(16) AML studies [3, 5–8, 10, 17, 39, 42] (654 patients); the pooled ORs were 1.42 (95% CI: 0.81–2.49, *P* = 0.48, *I*^*2*^ = 0%) and 0.95 (95% CI: 0.52–1.74, *P* = 0.48, *I*^*2*^ = 0%)l. These data indicate that the *KIT* mutations did not affect CR in inv(16)AML, and they had a mild but statistically non-significant effect on CR in t(8,21) AML. These results are consistent with those of previously published cohort studies [6, 8, 17, 39]. There were no heterogeneous data in each subgroup, and the Begg’s and Egger’s tests revealed no publication bias (Table 2).

**Fig 2:**
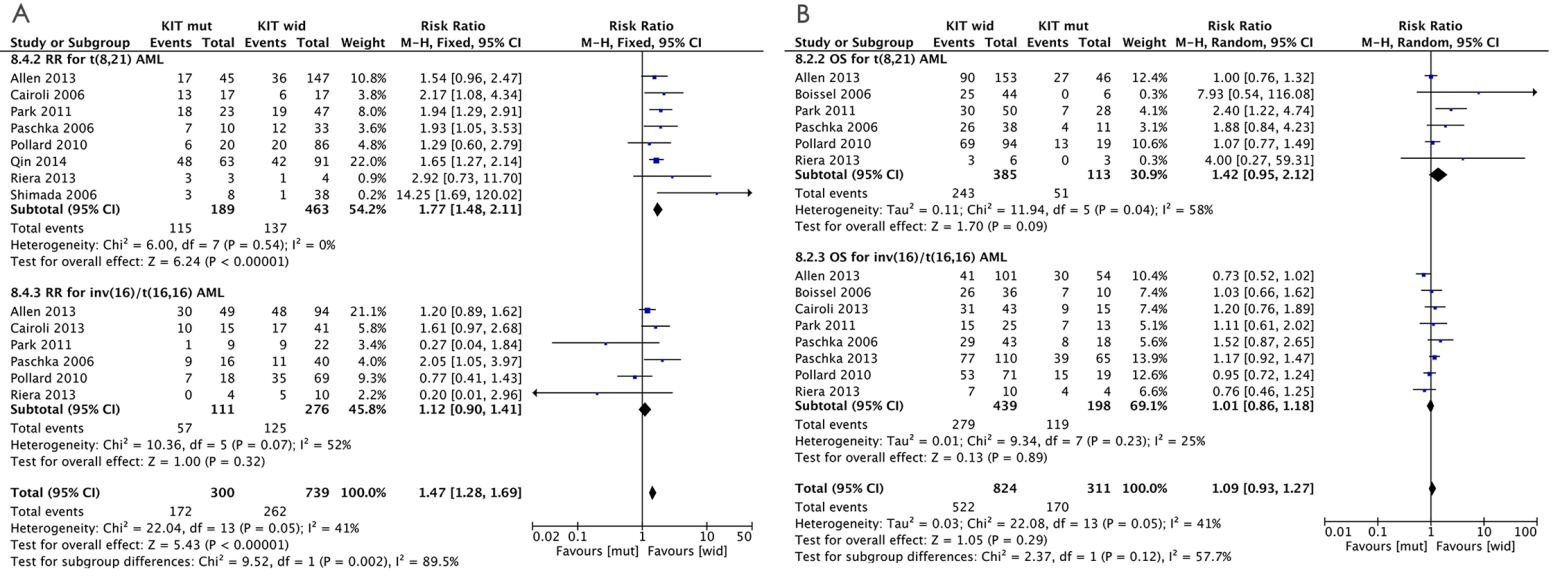
RRs and 95% CIs for (A) 2-year relapse risk and (B) 5-year OS, according to a subgroup analysis of CBF-AML. The number of included studies, number of patients in the included studies, and percentage of patients with *KIT* mutations in the included studies are listed.

For the subgroup analysis of relapse risk, we used eight t(8,21) AML studies [4, 5, 7, 8, 10, 29, 39, 42] (619 patients), and we used six inv(16) AML studies [5, 7, 10, 17, 39, 42] (387 patients) to evaluate the pooled RRs of relapse risk. Of the t(8,21) AML studies, two provided 2-year relapse data [8, 29], one provided 4-year relapse data [4], and five provided >5-year relapse data. As with the earlier analysis of the pooled RRs of CBF-AML, we also separately pooled the RRs for 2- and 5-year relapse risks, which were 1.76 (95%CI: 1.45–2.12; *P* = 0.52, *I*^*2*^ = 0%) and 1.70 (95% CI: 1.31–2.21; *P* = 0.78, *I*^*2*^ = 0%), respectively. These results suggest a significant *KIT* mutation-related increase in the relapse risk of t(8,21) AML. There was no evidence of heterogeneity and no publication bias across studies (Table 2). All six inv(16) AML studies provided >5-year data for relapse risk; the 5-year relapse pooled RR was 1.12 (95%CI: 0.90–1.41; *P* = 0.07, *I*^*2*^ = 52%; fixed effect model) and 1.18 (95% CI: 0.79–1.76; *P* = 0.07, *I*^*2*^ = 52%; random-effects model). Moderate heterogeneity was observed across these studies; publication bias was detected with the Begg’s and Egger’s tests, and the *P*-values were *P* = 1.000 and *P* = 0.324, respectively, suggesting a low probability of publication bias. Trim-and-fill analysis detected one imputed study, which would not have affected our results. In the “leave-one-out” sensitivity analysis of this subgroup, omitting any single study did not lower the *I*^*2*^ further than 47%, with no significant changes in the estimated RRs. These results suggest that, unlike t(8,21) AML, there was no significant *KIT* mutation-related increase in relapse risk of inv(16) AML. In addition, when we focused on the 5-year relapse risk, analysis of the subgroup differences between inv(16) and t(8,21) AML showed that *P* = 0.02 and *I*^*2*^ = 82% in the fixed-effects model, and *P* = 0.1 and *I*^*2*^ = 63% in the random-effects model. Based on the subgroup difference, it appears that when *KIT* mutations are considered, the CBF-AML relapse rate should not be evaluated together but in subgroups. Fig 2A shows the forest plot of OS in the subgroup meta-analysis.

For the subgroup analysis of OS, six t(8,21) AML studies (498 patients)[3, 5, 7, 10, 39, 42], and eight inv(16) AML studies (637 patients) [3, 5–7, 10, 17, 39, 42] were analyzed. The pooled RR of OS for t(8,21) AML was 1.35 (95% CI: 1.09–1.66; *P* = 0.04, *I*^*2*^ = 58%) in the fixed-effects model and 1.42 (95% CI: 0.95–2.12; *P* = 0.04, *I*^*2*^ = 58%) in the random-effects model. Moderate heterogeneity was found in these studies; we conducted publication bias analysis, and the Begg’s and Egger’s tests *P*-values were *P* = 0.452 and *P* = 0.023, respectively; the Egger’s test *P*-value indicated publication bias. We used the trim-and-fill method to calculate an adjusted pooled random-effects RR to evaluate the influence of potential publication bias. This method added three estimates to balance the funnel plot (S1 Fig), and the adjusted risk estimates were 1.071 (95% CI: 0.889–1.291) and 1.134 (95% CI: 0.761–1.688) in the fixed- and random-effects models, respectively, which suggested an undefined impact of the *KIT* mutations on the OS of t(8,21) AML. For the inv(16) AML subgroup, the pooled RRs of OS were 1.03 (95% CI: 0.90–1.18; *P* = 0.23, *I*^*2*^ = 25%) and 1.01 (95% CI: 0.86–1.18; *P* = 0.23, *I*^*2*^ = 25%) in the fixed- and random-effects models, respectively, suggesting no significant *KIT* mutation-related decrease in the OS of inv(16) AML. No significant heterogeneity and publication bias were found across the inv(16) AML studies, and the Egger’s and Begg’s tests *P*-values were *P* = 0.902 and *P* = 0.904, respectively. In addition, a subgroup difference between inv(16) and t(8,21) AML for OS was observed as *P* = 0.12 and *I*^*2*^ = 58% in the random-effects model and *P* = 0.03 and *I*^*2*^ = 78% in the fixed-effects model, which indicated that, as with the relapse rate, OS of CBF-AML should be evaluated in subgroups with in terms of the *KIT* mutations. Fig 2B shows a forest plot of relapse risk in the meta-analysis subgroup.

### Subgroup analysis according to ethnicity

Marcucci *et al*. [2] reported that non-Caucasians with t(8,21) AML failed induction chemotherapy more often (*P* = 0.006) and with shorter OS than Caucasians when certain secondary cytogenetic abnormalities were present, indicating that ethnicity is an important predictor in t(8,21) AML. In addition, the pooled RRs for OS of t(8,21) AML in the present study were not stable; therefore, we performed a subgroup analysis of t(8,21) AML according to race. We subdivided patients with t(8,21) AML into Caucasian and non-Caucasian subgroups, and the impact of *KIT* mutations on CR, relapse risk, and OS of the subgroups was analyzed separately based on the presence or absence of *KIT* mutations (Table 3). For CR, four studies [3, 5, 7, 39] with 411 Caucasian patients and five studies [4, 8, 10, 29, 42] with 315 non-Caucasian patients were included, respectively, and the respective pooled ORs were 0.61 (95% CI: 0.19–1.95, *P* = 0.73, *I*^*2*^ = 0%) and 2.03 (95% CI: 1.02–4.05, *P* = 0.41, *I*^*2*^ = 0%), suggesting that the *KIT* mutation has an adverse impact on the CR of non-Caucasian patients but not on that of Caucasian patients. No heterogeneous data were observed. For relapse risk in non-Caucasians, two studies provided 2-year relapse data [8, 29], and one provided 4-year relapse data [4]. Therefore, separately pooled RRs for 2-, 4-, and 5-year relapses were calculated as 1.89 (95% CI: 1.51–2.37; *P* = 0.26, *I*^*2*^ = 24%), 2.33 (95% CI: 1.59–3.41; *P* = 0.16, *I*^*2*^ = 45%), and 2.03 (95% CI: 1.37–3.01; *P* = 0.58, *I*^*2*^ = 0%), respectively. However, in Caucasians, three studies only provided a 5-year relapse data [5, 7, 39], and the pooled RR was 1.55 (95% CI: 1.10–2.18, *P* = 0.70, *I*^*2*^ = 0%). These data indicated a significant *KIT* mutation-related increase in relapse risk in both subgroups. As for OS, three studies [3, 5, 7] with 362 Caucasians and three studies [10, 39, 42] with 136 non-Caucasians were included; the pooled RRs were 1.12 (95% CI: 0.90–1.40, *P* = 0.26, *I*^*2*^ = 27%) and 2.26 (95% CI: 1.35–3,78, *P* = 0.82, *I*^*2*^ = 0%), respectively. No heterogeneous data were observed. These OS-related results showed that, similar to the CR rate but unlike the relapse risk, the *KIT* mutations adversely affected the OS of the non-Caucasian patients but not that of the Caucasian patients.

**Table 3:** Meta-analysis results by ethnicity.

Outcome	Ethnicity	N^^^	Patients, n	Mut^&^, (%)	Fixed	Random	Heterogeneity		Subgroup Differences			
AML					RRs^*^ 95%CI	RRs 95%CI	*P*	*I^2^*	Fixed		Random	
									***P***	***I^2^***	***P***	***I^2^***
**CR**												
t(8,21)												
	Caucasian	4	411	82(20)	0.61 [0.19, 1.95]	0.70 [0.21, 2.30]	0.73	0%				
	non-Caucasian	5	315	115(37)	2.03 [1.02, 4.05]	2.06 [1.01, 4.22]	0.41	0%	0.08	67%	0.13	57%
inv(16)												
	Caucasian	5	527	166(31)	0.80 [0.41, 1.59]	0.87 [0.43, 1.77]	0.51	0%				
	non-Caucasian	4	127	40(31)	1.96 [0.50, 7.72]	2.13 [0.47, 9.68]	0.31	3%	0.25	23%	0.30	9%
**OS**												
t(8,21)												
	Caucasian	3	362	71(20)	1.12 [0.90, 1.40]	1.06 [0.80, 1.40]	0.26	27%				
	non-Caucasian	3	136	42(31)	2.26 [1.35, 3.78]	2.22 [1.33, 3.70]	0.82	0%	0.01	84%	0.01	84%
inv(16)												
	Caucasian	5	527	166(31)	1.02 [0.88, 1.18]	1.01 [0.83, 1.25]	0.12	45%				
	non-Caucasian	3	110	32(29)	1.08 [0.79,1.47]	1.01 [0.74, 1.36]	0.34	7%	0.75	0%	0.97	0%
**Relapse Risk**												
t(8,21)												
5y	Caucasian	3	341	75(22)	1.55 [1.10, 2.18]	1.60 [1.14, 2.23]	0.70	0%				
2y	non-Caucasian	5	278	96(35)	1.89 [1.51, 2.37]	1.91 [1.41, 2.57]	0.26	24%				
4y	non-Caucasian	3	123	34(28)	2.33 [1.59, 3.41]	2.87 [1.15, 7.15]	0.16	45%				
5y	non-Caucasian	2	77	26(34)	2.03 [1.37, 3.01]	2.00 [1.35, 2.96]	0.58	0%	0.34	0%	0.44	0%
inv(16)												
5y	Caucasian	3	286	83(29)	1.18 [0.92, 1.52]	1.22 [0.78, 1.90]	0.11	55%				
	non-Caucasian	3	101	28(28)	0.94 [0.56, 1.58]	0.57 [0.10, 3.45]	0.07	52%	0.44	0%	0.43	0%

^^^ N: Studies included.

^*^ ORs for CR, and RRs for OS and relapse rate.

^&^ Numbers of patients with *KIT-*mutations.

The *KIT* mutations did not affect inv(16) AML, and ethnicity did not affect the clinical outcomes in this subgroup, nevertheless, we also performed the relevant analysis for the inv(16) AML subgroup. No significant effects of race were observed (Table 3).

### Association between *KIT* mutation genotypes and CBF-AML

Different *KIT* mutation locations have been reported in the CBF-AML and t(8;21) and inv(16) AML subgroups, which was insufficient for the meta-analysis but still systematically reviewed (Table 4). Exon 8 and 17 mutations were the two most-studied genotypes. The occurrence rates of the exon 8 and 17 mutations were 1.5–18.5% [14, 38] and 4–31% [3, 10], respectively, when CBF-AML was regarded as an group, and they were 0–13% [4]^,^[38] and 6–38% [3]^,^[29], respectively, in t(8;21) AML and 3–33% [17, 41] and 2–32%[3, 8], respectively, in inv(16) AML. Clearly, the exon 17 mutations were more common than the exon 8 mutations in t(8;21) AML, but the occurrence rates of these mutations appeared to be similar in inv(16) AML. S2, S3 and S4 Figs depict the distribution patterns of the *KIT* mutations.

**Table 4:** Studies assessing the prognostic relevance of *KIT* mutations in CBF-AML and subgroups in a systematic review.

Reference	N	Age range, y,(median)	Analyzed *KIT* exons	Proportion of patients with *KIT* mutations^a^, %	Median follow-up, y	Prognostic relevance of *KIT* mutations
**CBF AML**						
Jung, 2014^$^	75	18-75(NR)	8, 10, 11, 17	19(5/27)	NR	No impact on LFS or OS.
Jourdan, 2013^$^	198	18-60(42)	8, 17	20(40/198)	2.7	No impact on HR for relapse (UVA).
Markova, 2009^$^	60	29.3(1.6–72.2)	8, 9, 10, 11, 17, 18	47(28/60)	2.3	No impact on RR or OS.
Wang, 2012^$^	76	NR	8, 17	29(22/76)	NR	Inferior OS and RFS (MVA).^c^
Goemans, 2005^$^	27	NR	8, 9, 11, 17	63(17/27)	NR	No impact on DFS or EFS.
Allen, 2013	354	NR (adult) ^#^	8, 9, 10, 11, 17, 18	28(100/354)	8.3	Inferior 10-y CIR, no impact on 10-y OS.
Riera, 2013^b^	23	42.7 (19–64)	8, 9, 10, 11, 13, 14, 17	30(7/23)	3.7	No impact on DFS or OS.
Pollard, 2010	203	11.8(0.6–20)	8, 17	18(38/203)	5.5	No impact on RR, EFS, DFS or OS.
Kim, 2013^*^^,^^d^	121	41(15–71)	8, 10, 11, 13, 17	26(32/121)	2.3	Inferior 2-y EFS or 2-y OS (D816 V).
**t(8,21) AML**						
Jourdan, 2013^$^	96	18-60(42)	8, 17	23(22/96)	2.7	No impact on HR for relapse (UVA).
Jones, 2010^$^	82	39.6 (4–72)	8, 17	20(12/60)	2.3 ^f^	No impact on PFS or OS.
Markova, 2009^$^	34	29.3(1.6–72.2)^e^	8, 9, 10, 11, 17, 18	21(7/34)	2.3 ^f^	No impact on RI, OS was seemingly inferior to unmuted ones (p = 0.14).
Shih, 2008^$^	28	<17(NR)	8, 17	43(12/28)	NR	No impact on RR, EFS or OS.
Schnittger, 2006^$^	64	15-90(NR)	17	13(8/64)	NR	Inferior EFS and OS significantly in patients with *KIT* exon 17 mutations.
Qin, 2014	253	NR	8, 17	39(99/253)	0.83	Inferior on CIR, DFS, and OS with *KIT* mutation, particularly in *KIT* exon 17 mutations.
Riera, 2013^b^	9	NR	8, 9, 10, 11, 13, 14, 17	33(3/9)	3.7	No impact on DFS or OS.
Park, 2011	78	NR	8, 17	41(30/78)	NR	Inferior 5-y EFS or 5-y OS.
Pollard, 2010	113	11.8(0.6–20)^e^	8, 17	17(19/113)	5.5	No impact on RR, EFS, DFS or OS.
Boissel, 2006	56	NR	8, 17	12(6/50)	4.4	Inferior OS, RFS, and EFS
Paschka, 2006	49	NR (adult)	8, 17	22(11/49)	5.3	Inferior CIR with *KIT* mutation, particularly in *KIT* exon 17 mutations.
Shimada, 2006	46	7.5years (2–15)	8, 9, 10, 11, 17, 18	17(8/46)	NR	Inferior OS, DFS, and RR(exon17).
Cairoli, 2006	42	40.5(16–76)	8, 17	53(19/36)	2 ^g^	Inferior OS, RI, particularly for patients with *KIT* exon 17 mutations at codon D816.
Allen, 2013	199	NR (adult)^#^	8, 9, 10, 11, 17, 18	23(46/199)	8.3	Inferior 10-y CIR, no impact on 10-y OS.
Kim, 2013^*^^,^^d^	82	44(15–71)	8, 10, 11, 13, 17	NR	2.3	Inferior EFS or OS, for patients with *KIT* D816.
Krauth, 2014^*^	139	53.3(18.6–83.8)	8, 9, 10, 17	17(23/139)	2.2	Inferior 2-y OS and 2-yEFS with *KIT*D816.
Care, 2003	47	NR	8, 17	13(6/47)	3.8	NR
**inv(16) AML**						
Schwind, 2013^h^^,^ ^$^	208	41(17–73)	8, 17	18(39/208)	NR	Inferior OS and EFS.
Jourdan, 2013^$^	102	18-60(42)^e^	8, 17	18(18/102)	2.7	No impact on HR for relapse (UVA).
Jones, 2010^$^	94	33.8 (10 to 77)	8, 17	12(7/57)	2.3^d^	No impact on PFS or OS.
Markova, 2009^$^	26	29.3(1.6–72.2)^e^	8, 9, 10, 11, 17, 18	42(11/26)	2.3^d^	No impact on RI or OS.
Shih, 2008^$^	13	<17(NR)	8, 17	38(5/13)	NR	No impact on EFS or OS.
Qin, 2014	98	NR	8, 17	30(29/98)	0.8	No impact on CIR, and OS, a tendency in inferior DFS.
Cairoli, 2013	58	42 (15–60)	2, 8, 10, 11, 17	25(15/58)	4.2	No impact on RI or OS.
Paschka, 2013	176	41(18–74)	8, 10, 11, 17	37(65/176)	6.04	Inferior RFS, not OS.
Park, 2011	38	NR	8, 17	34(13/38)	NR	No impact on EFS or OS.
Pollard, 2010	90	11.8(0.6–20)^e^	8, 17	21(19/90)	5.5	No impact on RR, EFS, DFS or OS.
Boissel, 2006	47	NR	8, 17	22(10/46)	4.4	No impact in OS, RFS, and EFS.
Paschka, 2006	61	NR (adults)	8, 17	30(18/61)	5.3	Inferior CIR, patients with *KIT* mutation, particularly in *KIT* exon 17 mutations, and inferior OS, patients with *KIT* mutation, in *KIT* exon 8 or 17 mutations.
Cairoli, 2006	25	51(17–88)	8, 11	47(8/17)	2^g^	No impact on RI or OS.
Allen, 2013	155	NR (adult)^#^	8, 9, 10, 11, 17, 18	35(54/155)	8.3	No impact on CIR, OS.
Kim, 2013^*^^,^^d^	39	38(18–69)	8, 10, 11, 13, 17	NR	2.4	No impact on EFS or OS.
Care, 2003	63	NR	8, 17	32(20/63)	3.8	Inferior relapse rate in patients with *KIT* exon 8 mutations, but not in OS.

*Studies for sensitivity analysis.

^#^ Age range was 15–59 years, n = 335; > 60, n = 19.

^$^ Studies included in the systematic review and excluded from the meta-analysis.

^a^ Number of patients with *KIT* mutations/patients checked with *KIT* mutation status studied.

^b^ Data obtained from the corresponding author.

^c^ Adjustment details for MVA were not reported.

^d^ Only evaluated the effect of the *KIT* D816 mutation in CBF-AML.

^e^ Median age provided for CBF-AML as an entity.

^f^ Median follow-up provided CBF-AML as an entity.

^h^ Divided into type A and non–type A inv(16) AML; *KIT* mutations shown as an important prognosticator in type A inv(16) AML, not in non–type A inv(16) AML.

^g^ Median follow-up for OS and RI was 2 and 1.3 years, respectively.

Abbreviations:

NR, not reported

RR, relapse rate

HR, hazard ratio

PFS, progression-free survival

CIR, cumulative incidence of relapse

RI, relapse incidence

OS, overall survival

EFS, event-free survival

RFS: relapse-free survival

DFS, disease-free survival

UVA, univariate analysis

MVA, multivariable analysis.

For the prognosis analyses of the 23 studies [3–17, 29, 38–44] included in this systematic review, 12 studies [4, 7–9, 11, 12, 14, 15, 29, 39, 40, 44] reported *KIT* mutation location data. Jourdan *et al*. [15] showed that the OS and recurrence-free survival rate in patients with CBF-AML with exon 8 or 17 mutations did not significantly differ from those in patients without *KIT* mutations, regardless of CBF-AML subtype, whereas Paschka *et al*. [39] reported that both exon 8 and 17 mutations, particularly exon 17, adversely affected the 5-year OS and relapse risk of inv(16) AML. Seven studies showed that exon 17 mutations adversely affected the OS and other clinical outcomes of t(8,21) AML [4, 8, 11, 12, 29, 39, 40]. Cairoli *et al*. [17] found that exon 17 mutations, particularly at codon D816, adversely affected the OS and relapse risk of patients with t(8;21) AML but not that of patients with inv(16) AML, which was consistent with the findings of two recent studies [11, 12]. However, Allen *et al*. [5] and Pollard *et al*. [7] found that *KIT* mutations resulted in similar prognostic effects regardless of *KIT* genotype. The lack of detail in investigations based on *KIT* genotypes in other studies may have resulted from the limited sample size.

### Association between *KIT* mutations and pediatric CBF-AML

In this systematic review, six included studies [4, 7, 9, 13, 29, 38] analyzed data or descriptions of pediatric patients (< 17 years) with CBF-AML. Four studies [4, 7, 13, 38] focused on pediatric CBF-AML; two studies [9, 29] partly described and analyzed pediatric patient data. Of the six studies, four [7, 9, 13, 29, 38] found that *KIT* mutations did not significantly impact the prognosis of relapse risk, EFS, DFS, or OS, which was consistent with the findings in the largest study reported by Pollard *et al*. [7], which involved 203 pediatric patients with CBF-AML. However, Shimada *et al*.[4] and Manara *et al*.[31] (excluded from the systematic review because they were letters to the editor) both found that *KIT* mutations adversely affected the OS, DFS, or relapse rate of pediatric patients with t(8,21) AML. Manara *et al*. [31] found that *KIT* mutations demonstrated no significant impact on the prognosis of inv(16) AML; however, Shamda *et al*. [4] did not research or describe inv(16) AML. In the meta-analysis, omitting the two studies that focused on pediatric patients [4, 7] did not alter the results or reduce the heterogeneity (S4 Table). These findings indicate that the inconsistent reporting of *KIT* mutation-related risks in the prognosis of t(8,21) AML also exists in pediatric patients; the systematic review also implied that pediatric CBF-AML should not be treated as a group but rather as t(8,21) and inv(16) AML subgroups. Thus, these results are consistent with the results of our meta-analysis.

The systematic review revealed that in CBF-AML, there was a highly similar occurrence rate for *KIT* mutations in pediatric patients (19–44% [7, 29]) compared to that in overall patients (16–46% [8, 16]), and the rates were 17–42% [7, 29] and 21–55% [7, 38] for pediatric t(8,21) AML and inv(16) AML, respectively. *KIT* mutation genotypes in pediatric patients with CBF-AML showed a distribution pattern similar to that of the overall patient group. In pediatric patients, the occurrence rates of the exon 8 mutations were 10–19% [29, 38], 0–13% [4, 38], and 16–27% [7, 38] for CBF-AML, t(8,21) AML, and inv(16) AML, respectively. The occurrence rates of the exon 17 mutations were 8–34% [7, 29], 12–44% [7, 46], and 4–31% [7, 29] for CBF-AML, t(8,21) AML, and inv(16) AML, respectively. There appear to be no rules for *KIT* mutation genotype distribution. S5 Fig shows the distribution pattern of *KIT* mutations in pediatric CBF-AML.

### Sensitivity analysis

We conducted “leave-one-out” sensitivity analysis: one study included in the meta-analysis was evaluated each time to determine the influence of the individual data set on the pooled RRs, and omitting any single study caused no significant changes. In addition, after including three studies that provided 2-year OS data and two studies with 4-year OS [4, 11, 12, 29] for the pooled analysis of 2-year OS, the pooled RRs showed a minor variation similar to that of the pooled analysis of the 5-year OS, indicating that our results on the impact of *KIT* mutations on the OS of CBF-AML and its subgroups are fairly robust. Moreover, summarized subgroup analysis showed similar overall effects to that of the CBF-AML as an group, also suggesting that the meta-analysis results for the 5-year OS and relapse rate are robust.

## Discussion

This study reports the first meta-analysis (to our knowledge) evaluating the impact of *KIT* mutations on the prognosis of CBF-AML, summarizing the results of 11 studies involving 1 380 CBF-AML patients. Based on the current controversy regarding the prognostic significance of *KIT* mutations in CBF-AML, we primarily focused on CBF-AML as one group and sought to determine whether the clinical outcomes are associated with *KIT* mutations. We found that the *KIT* mutations had no effect on CR, as previously reported [8, 17, 39, 43], but they resulted in a significantly increased relapse risk. However, it has been reported that *KIT* mutations play different clinical roles in the inv(16) and t(8,21) AML subtypes [5, 6, 8, 9, 17, 39], and these two CBF-AML subtypes should be considered as distinct entities[2]. Therefore, we performed a subgroup meta-analysis of the clinical outcomes of these subtypes. The *KIT* mutations did not affect the CR of inv(16) or t(8,21) AML, and the *KIT* mutation-related relapse risk of t(8,21) AML was significantly increased, but it was not increased in inv(16) AML, indicating that the increased relapse risk of CBF-AML may be due to the risk of t(8,21) AML but not inv(16) AML. Furthermore, in inv(16) AML, the OS was not significantly affected by the *KIT* mutations; however, the OS tended to be shorter in t(8,21) AML patients with *KIT* mutations. To date, inv(16) and t(8,21) AML have been considered to have relatively favorable prognoses and to have been treated similarly. However, with the distinct effects of *KIT* mutations in our subgroup analyses, CBF-AML with *KIT* mutations should be regarded as distinct and heterogeneous entities with different outcomes.

Because the meta-analysis result of the OS in t(8,21) AML was not definitive, using race as a risk factor in t(8,21) AML [2], we subdivided t(8,21) AML into Caucasian and non-Caucasian subgroups for analysis. Surprisingly, *KIT* mutations were found to adversely impact the CR of non-Caucasian patients but not that of Caucasian patients, a finding that has never been reported and is consistent with the findings of a study involving non-Caucasian patients [9]. Moreover, the *KIT* mutations increased the relapse risk in both non-Caucasian and Caucasian patients, and they adversely affected the OS of non-Caucasian patients but not that of Caucasian patients. A study by Marcucci *et al*. also reported that the nonwhite patients with t(8;21) have higher odds of failing induction therapy compared with the corresponding white patient population [2]. The underlying reasons for these findings may be attributed to the social issues such as unequal access to health care and compliance, or distinct ethnic genetic background, since no obvious differences in treatment were found between the two distinct ethnic groups in the present meta-analysis. Future trials should be done to define distinct patterns of genetic background that might elucidate the molecular bases for different outcomes. Our primary results indicate that t(8,21) AML patients with *KIT* mutations should be evaluated according to ethnicity, which is consistent with other studies [3, 6, 9, 11, 39] and partially explained the uncertain role of *KIT* mutations in OS of t(8,21) AML as an entity in the present analysis. A similar analysis for inv(16) AML revealed that ethnicity is not a risk factor for this CBF-AML subtype with *KIT* mutations; the potential risk factors require further investigation.

In contrast to adult CBF-AML patients, most pediatric studies did not show that the *KIT* mutations were of prognostic relevance [7, 13, 38]. Thus, being a child or adult is a confounding factor and should be considered for the present analysis. We excluded two pediatric studies [4, 7] from the meta-analysis and analyzed the results of nine studies; we found no changes to the findings for CR, relapse rate, and OS, which indicated that the pediatric studies did not affect our earlier conclusions. Moreover, our systematic review indicated a controversial role of *KIT* mutations in pediatric CBF-AML, which requires future confirmation.

In CBF-AML, *KIT* mutations occur mainly on exon 8 or 17, and it is unclear whether the prognostic significance differs according to the subtypes of CBF abnormalities [5, 7, 8, 12, 39], which we should have studied in the meta-analysis. However, the role of these genotypes was not analyzed due to the lack of sufficient data in the included studies. Meanwhile, the three largest and most recent studies [5, 7, 15] observed that *KIT* mutations should be considered as an entity, not in genotypes, for the prognostic effect in CBF-AML, which must be determined in further high-quality studies. Note that the detection method potentially influences the evaluated result in this study, as reported by Wakita *et al*. [47], and more accurate and sensitive detection methods are urgently needed.

There might be other associated predictors, such as differences in clinical characteristics, chemotherapy-related AML, transformed AML, WBC count, age and individualized treatment. Increasing age has been reported to be the best predictor for survival of CBF-AML patients aged less than or equal to 60 years at univariate and multivariate analysis [2,6,17]. In the present meta-analysis, though omitting the two studies [4, 7] that focused on pediatric patients did not alter the results or reduce the heterogeneity (S4 Table), the effect of increasing age on clinical outcome of CBF-AML with *KIT* mutations could not be technically analyzed. Allo-SCT, as in the included studies for the meta-analysis, was administrated in pediatric patients if a suitable donor was available [3, 4, 7], but the role of SCT on prognosis was not evaluated, owing to the low rate of *KIT* mutations in the reported patients and to the limited patients (with or without *KIT* mutations) received SCT. In adult patients, most studies excluded the patients who received SCT from the study or analysis [6, 8, 29, 42]. In one study [10], the patients received SCT at first establishment of CR status or later, depending on the patient’s age and availability of a suitable donor, but no differences were found in incidence of SCT in first CR, OS, EFS, time interval for relapse according to the mutation status of *KIT*. Another study [17] found SCT to improve outcome of relapse or refractory CBF-AML, but the mutation status of *KIT* was not considered, which was also reported by Zhu *et al* [48]. Thus, the effect of SCT on prognosis of CBF-AML with *KIT* mutations has not yet been determined. As for *FLT*3 mutations, the potentially risky mutations in CBF-AML, they have been evidenced to negatively affect clinical outcome of CBF-AML [3, 5, 6, 38, 41], and the patients with *FLT*3 and *KIT* mutations have a even worse prognosis [6]. However, in this meta-analysis, limited to the available data, the role of *FLT*3 mutations in the outcomes of CBF-AML with *KIT* mutations could not be analyzed or determined. More substantial studies are necessary. For other mutations, such as *RAS*, *JAK*2, *IDH* and *WT1*, no prognostic significance has been found in CBF-AML [3, 5, 6, 9, 11, 38, 41, 49]. Meanwhile, in this analysis, most studies showed that the clinical characteristics were relatively homogeneous [7, 17, 38, 42]. Early in 2003, Cairoli *et al*.[32] reported that *KIT* mutations were associated with higher WBC counts, which subsequent studies did not support [6, 10, 17]. The debate is ongoing. In a recent study, patients with inv(16) AML with both *KIT* and *FLT3* mutations had significantly higher WBC counts compared to patients without the mutations [6]. In addition, we performed another meta-analysis and found that *KIT* mutations were significantly associated with higher WBC counts in inv(16) AML but not in t(8;21) AML (data not shown). In the present analysis, however, the *KIT* mutations demonstrated obvious effects on the clinical outcomes of t(8;21) AML patients. These findings suggest that higher WBC counts are not a confounding risk factor for our *KIT* mutation-related analyses of the clinical outcomes of t(8;21) AML patients.

One limitation of this meta-analysis is that we used data that were abstracted from published reports, and we performed study-level analysis; the substantial effect of heterogeneity must be considered. There are other potential limitations. First, observational prospective studies for a rare disease are logistically difficult to conduct, and the observational nature of the available studies renders such studies unavoidably open to the influence of residual confounders. Second, a relatively small number of studies could be included. Third, *KIT* transcript expression levels in CBF-AML were not considered, although Allen *et al*. reported that the levels of *KIT* mutations are clinically meaningful for prognoses of CBF-AML [5]. Fourth, the included studies differed in their detection quality of *KIT* mutations, which may have also resulted in data heterogeneity. Finally, publication bias is inevitable, although we did not detect it directly, and it has been reported that the occurrence of t(8,21)AML and inv(16) AML differ according to geographical location [9]. In addition, language bias may have occurred because we excluded articles that were not written in English, although all eligible articles with large cohorts from a wide range of non-English-speaking countries across Europe and Asia were included.

Despite these limitations, we specifically evaluated the relevance of *KIT* mutations to CBF-AML, performing subgroup analysis according to CBF abnormalities and patient ethnicity, thereby rendering this meta-analysis more powerful than any individual study. Based on the current evidence, these data present the most comprehensive view, to date, of the prognostic significance of CBF-AML. Additionally, we systematically reviewed the summarized association between *KIT* mutation genotypes and CBF-AML, and *KIT* mutations and pediatric CBF-AML. We did not exclude any article during the identification and selection process, and only the *P*-value of the Egger’s test indicated publication bias in terms of OS for t(8,21) AML as an entity. Similarly, the heterogeneity tests indicated little variability between studies that cannot be explained by chance. Moreover, we have performed several sensitivity analyses to examine the potential sources of heterogeneity and to evaluate robustness in the subgroups. In conclusion, this study indicates that *KIT* mutations are the key risk factors for the prognosis of t(8,21) AML but not inv(16) AML, thereby supporting the inclusion of testing for *KIT* mutational status in the initial routine diagnostic workup and stratification system of t(8,21) AML. We also demonstrated the negative role of ethnicity (i.e., for non-Caucasians but not for Caucasians) in the CR and OS of t(8,21) AML with *KIT* mutations. These results support the early prediction of the worse prognoses, as well as effective minimal residual disease monitoring of patients with t(8,21) AML with *KIT* mutations. It would be valuable to include *KIT* mutations, even the genotypes, as prognostic factors for risk prediction in prospective large-scale clinical trials. Until data from these future studies become available, the present analysis helps to define the prognostic significance of *KIT* mutations in CBF-AML, particularly the t(8,21) AML subgroup.

## Supporting Information

S1 Fig Filled funnel plot with pseudo 95% confidence limits. (PDF)

S2 Fig The distribution pattern of *KIT* mutations in CBF-AML. (PDF)

S3 Fig The distribution pattern of *KIT* mutations in t(8,21)-AML. (PDF)

S4 Fig The distribution pattern of *KIT* mutations in inv(16)-AML. (PDF)

S5 Fig The distribution pattern of *KIT* mutations in pediatric CBF-AML. (PDF)

S1 Table Treatment characteristics of included studies and detection method used for *KIT* mutations. (PDF)

S2 Table NOS for assessing the quality of included studies. (PDF)

S3 Table Outcomes in CBF-AML according to *KIT* mutations. (PDF)

S4 Table Meta-analysis results for adults. (PDF)

S5 Table Meta-analysis on Genetic Association Studies Checklist. (DOCX)
