# Supplementary figures and images for: Prognostic Significance of KIT Mutations in Core-Binding Factor Acute Myeloid Leukemia: A Systematic Review and Meta-Analysis
Source: PLoS One. 2016 Jan 15;11(1):e0146614. doi: 10.1371/journal.pone.0146614 (PMC4714806; doi:10.1371/journal.pone.0146614)

Filled funnel plot with pseudo 95% confidence limits

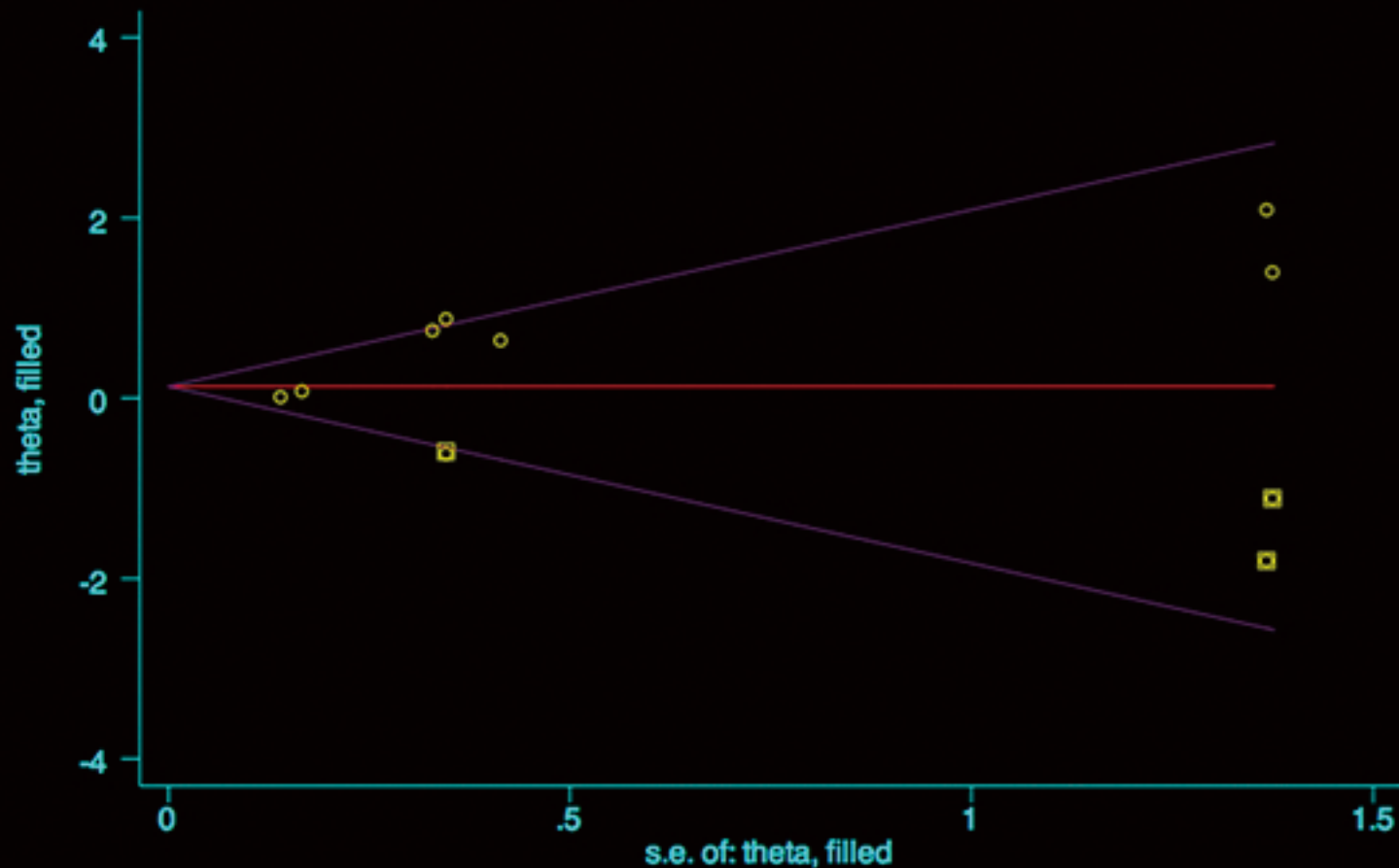

Supplement: S1 Fig — (PDF) [file pone.0146614.s001.pdf]

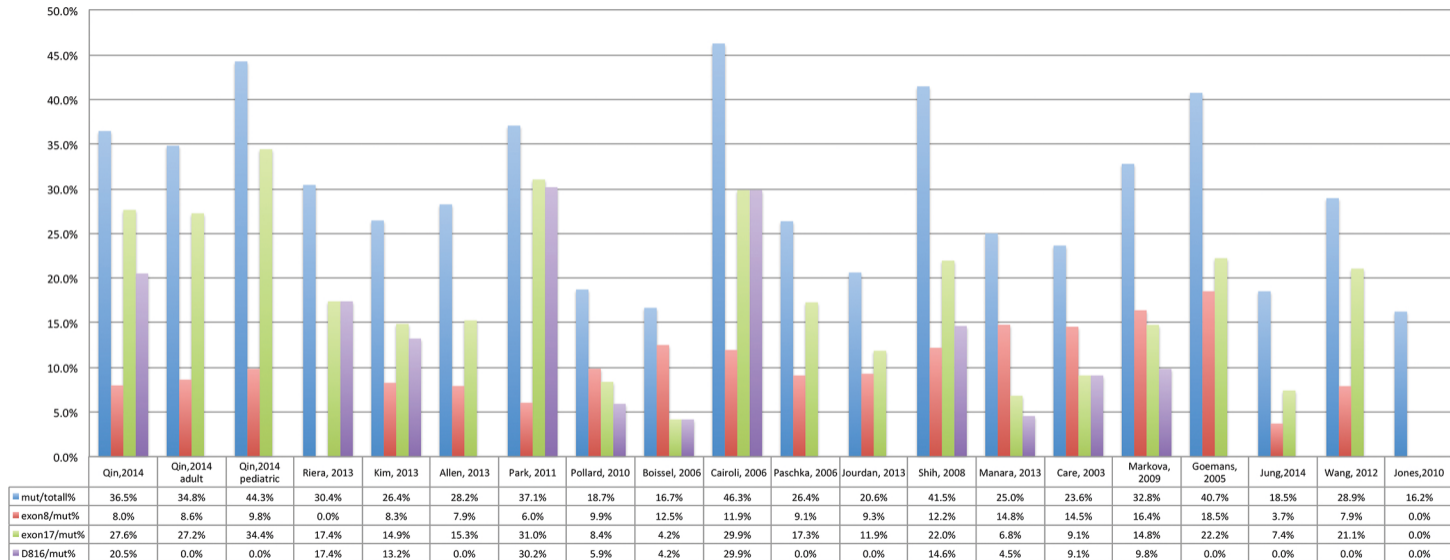

Supplement: S2 Fig — (PDF) [file pone.0146614.s002.pdf]

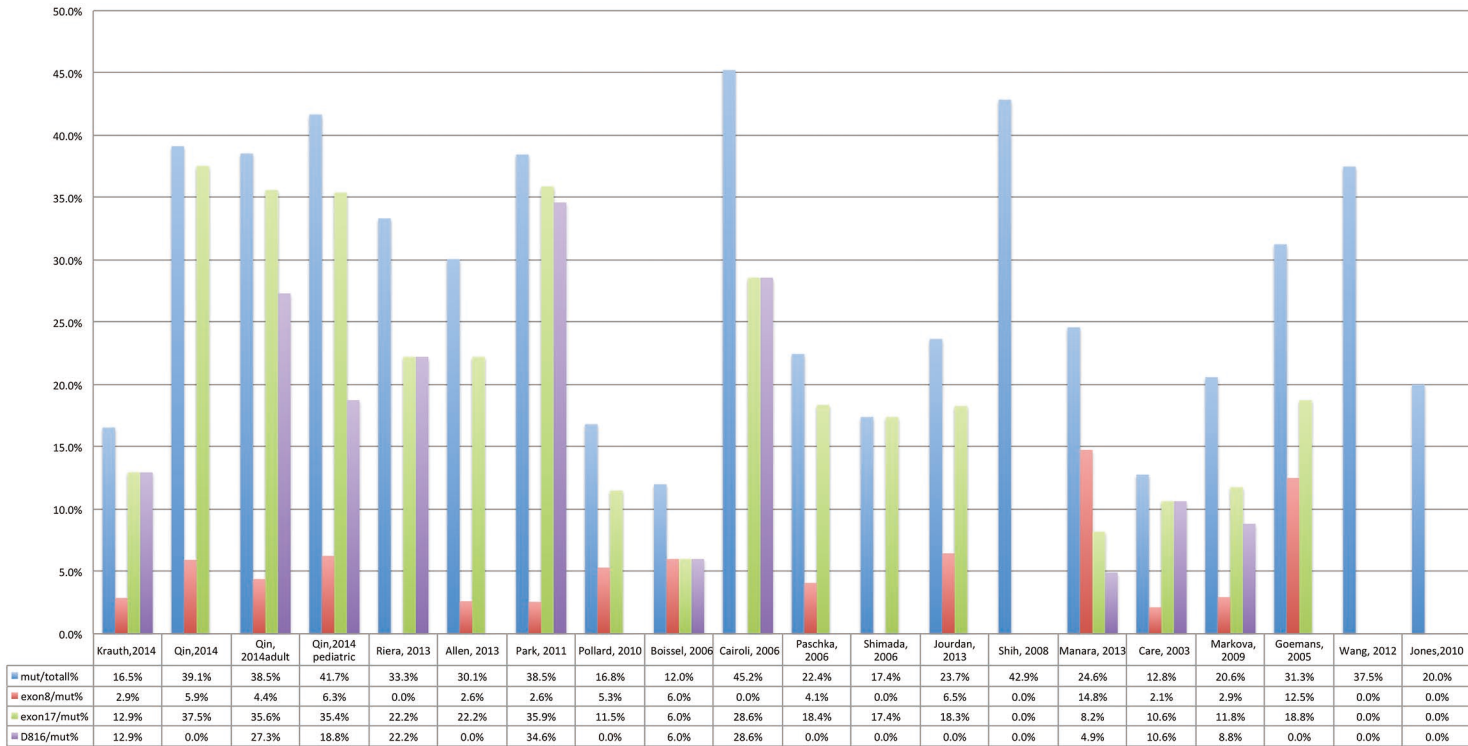

Supplement: S3 Fig — (PDF) [file pone.0146614.s003.pdf]

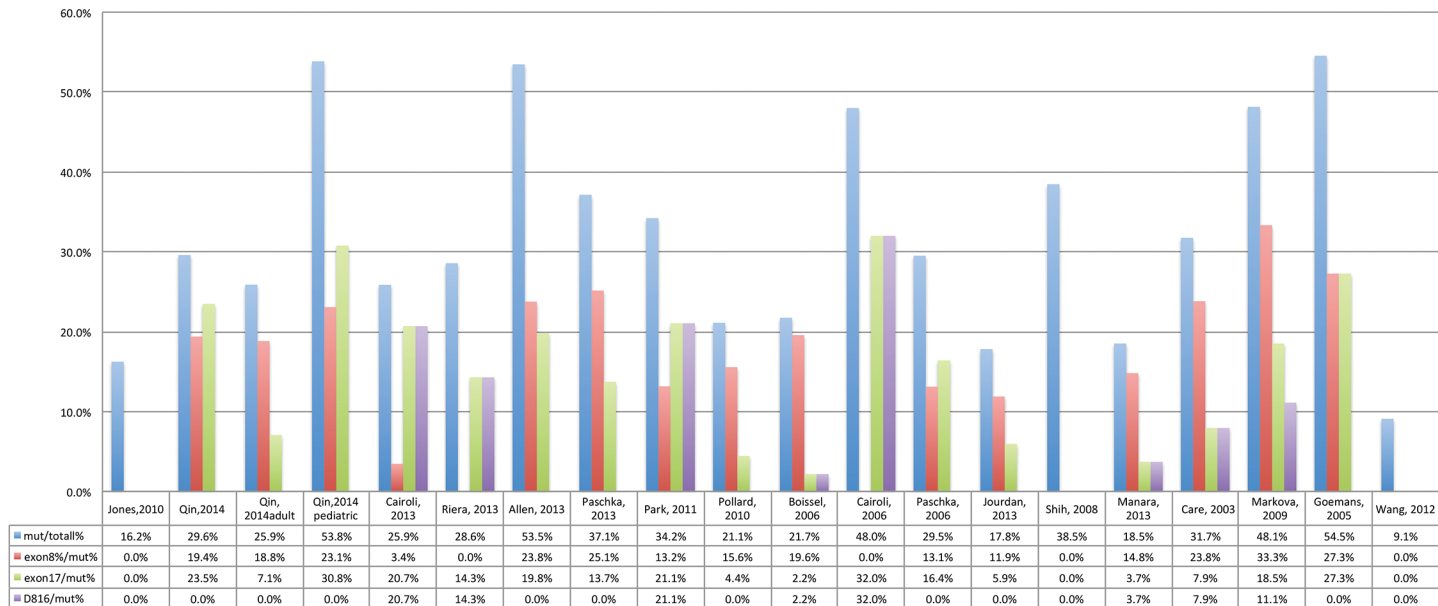

Supplement: S4 Fig — (PDF) [file pone.0146614.s004.pdf]

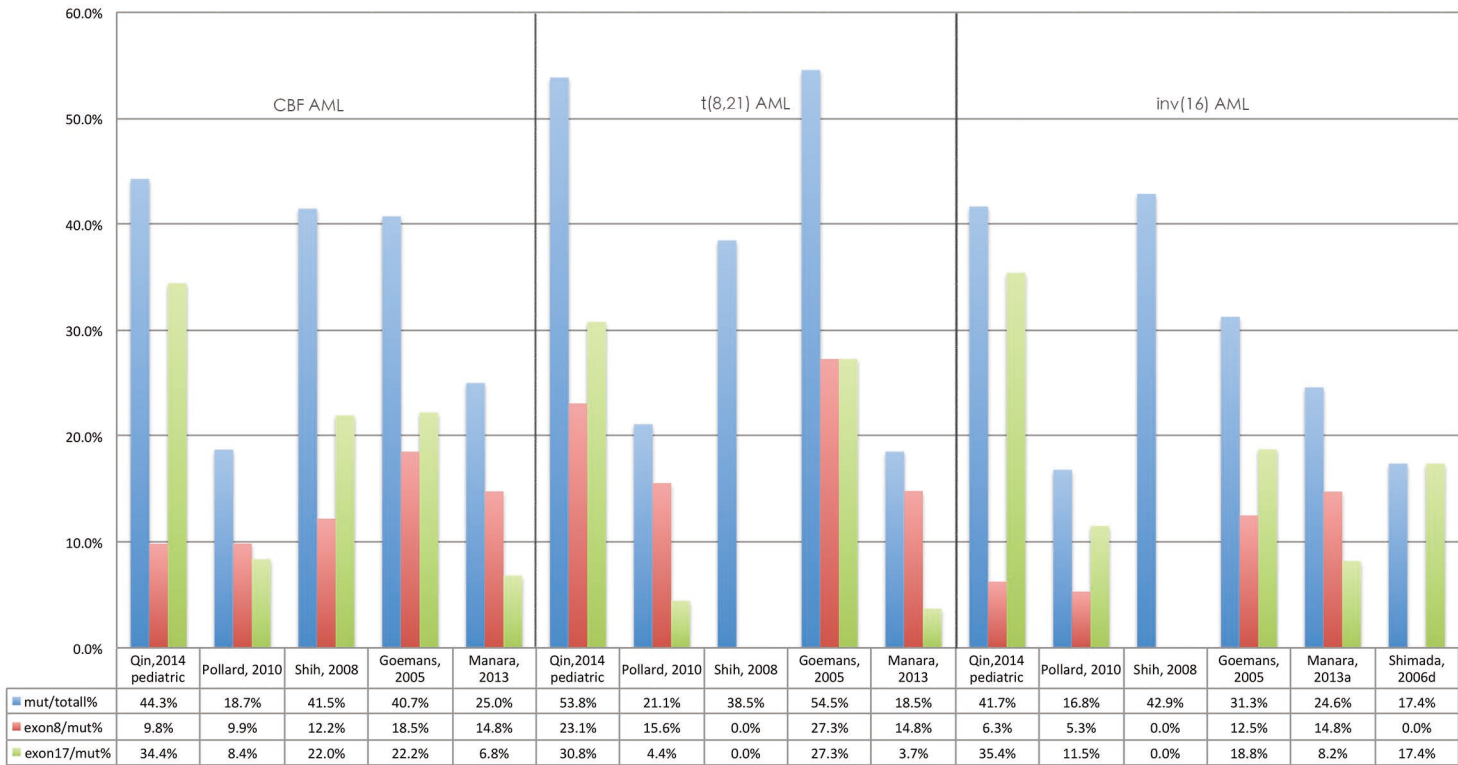

Supplement: S5 Fig — (PDF) [file pone.0146614.s005.pdf]
