# Supplementary material for: Prognostic Significance of KIT Mutations in Core-Binding Factor Acute Myeloid Leukemia: A Systematic Review and Meta-Analysis
Source: PLoS One. 2016 Jan 15;11(1):e0146614. doi: 10.1371/journal.pone.0146614 (PMC4714806; doi:10.1371/journal.pone.0146614)
Supplement: S1 Table — (PDF) [file pone.0146614.s006.pdf]

Reference	Induction Treatment	Post-Remission Chemotherapy	Detection Method
Allen，2013	UK MRC AML10, AML12 or AML15 trials	Details of the trial protocols have been published elsewhere.	PCR, Direct sequencing
Cairoli, 2006	DA"3+7" or ICE	3 consolidation courses include cytarabine	PCR, Direct sequencing
Boissel, 2006	According to previous or current adult ALFA (ALFA90, ALFA98) or pediatric LAME (LAME91, LAME99, ELAM02) trials	High-dose cytarabineals. Owing to the low rate of mutations reported in our study, we did not evaluate the place of allogeneic SCT performed in only 10% of patients (10/97 in first CR).	PCR, Direct sequencing
Paschka, 2006	DA"3+7" or ICE	3 or 4 consolidation courses include cytarabine	PCR, Direct sequencing
Pollard, 2010	Details of treatment protocols POG-9421, CCG-2891, and CCG-2961. COG AAML03P1	Details of treatment protocols POG-9421, CCG-2891, and CCG-2961	PCR, Direct sequencing
Shimada, 2006	ICE	5 additional courses of intensive chemotherapy (high-dose cytarabine [HDCA], etoposide, idarubicine, and mitoxantron).	PCR, Direct sequencing
Paschka, 2013	Intensive anthracycline /cytarabine-based induction therapy	Intensive post remission therapy.	PCR, Direct sequencing
Park, 2011	DA‘7 + 3’	Intermediate-dose cytarabine-based chemotherapy, chemotherapy with autologous- hematopoietic stem cell transplantation (HSCT), and chemotherapy with allogeneic (allo)-HSCT for consolidation.	PCR, Direct sequencing
Riera, 2013	ICE	Consolidation treatment with high-does cytarabine.	PCR, Direct sequencing
Qin, 2014	ICE or DA‘3 + 7’	Consolidation treatment with high-does cytarabine for adult, chemotherapy with allogeneic (allo)-HSCT for children.	PCR, bidirectional sequencing
Cairoli, 2013	DA‘3 + 7’	Three doses of anthracycline or the “ICE” schedule.	PCR, Direct sequencing

PCR: Quantitative Real-time, BHAC: behenoylcytosine arabinoside, DBHAC‘3 + 7’: regimen with three doses of anthracycline and behenoylcytosine arabinoside in 7 day continuous intravenous infusion, DA‘3 + 7’: regimen with three doses of anthracycline and cytarabine in 7 day continuous intravenous infusion, ICE: schedule including etoposide 100 mg/m2/day on days 1 – 5, TAD: 6-thioguanine, cytarabine and daunorubicin, HAM: high- dose cytarabine and mitoxantrone, HSCT: hematopoietic stem cell transplantation, SCT: stem cell transplantation
