# Supplementary material for: Prognostic Significance of KIT Mutations in Core-Binding Factor Acute Myeloid Leukemia: A Systematic Review and Meta-Analysis
Source: PLoS One. 2016 Jan 15;11(1):e0146614. doi: 10.1371/journal.pone.0146614 (PMC4714806; doi:10.1371/journal.pone.0146614)
Supplement: S2 Table — (PDF) [file pone.0146614.s007.pdf]

Study	Selection	Comparative	Outcome	Score
Cairoli, 2013	★★★★	★★	★★★	9/9
Riera, 2013	★★★★	★★	★★	8/9
Allen, 2013	★★★★		★★	6/9
Boissel, 2006	★★★★		★★	6/9
Paschka, 2006	★★★★		★★	6/9
Pollard, 2010	★★★★	★★	★★★	8/9
Shimada, 2006	★★★★	★★	★★	8/9
Paschka, 2013	★★★★	★	★★★	7/9
Cairoli, 2006	★★★★	★★	★★	8/9
Park, 2011	★★★★	★★	★★	8/9
Qin, 2014	★★★★	★★	★★	8/9
