# Supplementary material for: Prognostic Significance of KIT Mutations in Core-Binding Factor Acute Myeloid Leukemia: A Systematic Review and Meta-Analysis
Source: PLoS One. 2016 Jan 15;11(1):e0146614. doi: 10.1371/journal.pone.0146614 (PMC4714806; doi:10.1371/journal.pone.0146614)
Supplement: S3 Table — (PDF) [file pone.0146614.s008.pdf]

Authors	KIT status	N	CR rate	Relapse	OS	P-value
CBF-AML						
Riera, 2013	KIT wid	16	88%	38% 5y RR	63% 5y	0.9
	KIT mut	7	100%	43% 5y RR	57% 5y	
Allen, 2013	KIT wid	254	95%	35% 10y CIR	59% 5y	0.3
	KIT mut	100	94%	50% 10y CIR	61% 5y	
Pollard, 2010	KIT wid	153	94%	34% 5y RR	74% 5y	0.819
	KIT mut	37	97%	35% 5y RR	76% 5y	
Cairoli, 2013	KIT wid	43	95%	41.8% 5y RI	72.1% 5y	0.5691
	KIT mut	15	100%	65.5% 5y RI	62.3% 5y	
Paschka, 2013	KIT wid	110	88%	NR	70% 6y	0.49
	KIT mut	65	94%	NR	61% 6y	
Park, 2011	KIT wid	75	92%	41% 5y RR	60% 5y	NR
	KIT mut#	41	78%	58% 5y RR	34.14% 5y	
Cairoli, 2006	KIT wid	17	100%	35% 2y RR	76.5% 2y	0.017
	KIT mut	19	89%	77% 2y RR	42.1% 2y	
Shimada, 2006	KIT wid	38	100%	3% 4y RI	97.4% 4y	0.001
	KIT mut	8	100%	38% 4y RI	50% 4y	
Paschka, 2006*	KIT wid	81	90%	31.5% 5y RR	58.02% 5y	
	KIT mut	29	90%	62% 5y RR	41.37% 5y	
Qin, 2014[a]	KIT wid	106	86%	46.2% 2y CIR	65.7% 2y	0.0055
	KIT mut	79	79%	73.2% 2y CIR	48.9% 2y	
Kim, 2013*	KIT wid	72	93%	NR	71.2% 2y	0.305
	KIT mut	39	94%	NR	55.5% 2y	
	KIT mutD816	13	85%	NR	37%	
Krauth, 2014*	KIT wid	92	NR	NR	77.8% 27	
	KIT mutexon8	3	NR	NR	100% 2y	
Krauth, 2014*	KIT wid	80	NR	NR	82% 2y	0.03
	KIT mutD816	15	NR	NR	59.1% 2y	
inv(16) AML						
Cairoli, 2013	KIT wid	43	95%	41.8% 5y RI	72.1% 5y	0.5691
	KIT mut	15	100%	65.5% 5y RI	62.3% 5y	
Allen, 2013	KIT wid	101	93%	51% 10y CIR	59% 10y	NR
	KIT mut	54	91%	62% 10y CIR	45% 10y	
Paschka,2013	KIT wid	110	88%	NR	70% 6y	0.49

	KIT mut	65	94%	NR	61% 6y	
Riera, 2013	KIT wid	10	100%	50% 5y RR	73% 5y	0.28
	KIT mut	4	100%	0% 5y RR	100% 5y	
Park, 2011	KIT wid	25	88%	41% 5y RR	61.6% 5y	0.601
	KIT mut	13	69%	11% 5y RR	53.9% 5y	
Pollard, 2010	KIT wid	71	97%	51% 5y RR	75% 5y	0.607
	KIT mut	19	95%	38% 5y RR	81% 5y	
Boissel, 2006	KIT wid	36	84%	NR	74.3% 6y	0.75
	KIT mut	10	100%	NR	72% 6y	
Paschka, 2006	KIT wid	43	93%	28% 5y RR	68% 5y	0.12
	KIT mut	18	89%	56% 5y RR	48% 5y	
	KIT mut17	10	100%	80% 5y CIR	40% 5y	0.22
	KIT mut8	8	75%	17% 5y CIR	50% 5y	0.19
t(8;21) AML						
Allen, 2013	KIT wid	153	96%	25% 10y-CIR	59% 10y	0.9
	KIT mut	46	98%	38% 10y-CIR	60% 10y	
Riera, 2013	KIT wid	6	67%	17%	50% 5y	0.1
	KIT mut	3	100%	100%	0% 5y	
Pollard, 2010	KIT wid	94	92%	23%5y RR	74% 5y	0.603
	KIT mut	19	100%	31%5y RR	71% 5y	
Boissel, 2006	KIT wid	44	98%	NR	57.2% 6y	0.03
	KIT mut	6	83%	NR	0% 6y	
Paschka, 2006	KIT wid	38	87%	36%5y RR	48% 5y	0.49
	KIT mut	11	91%	70%5yRR	42% 5y	
Park, 2011[b]	KIT wid and 8	50	94%	40%5y RR	60.9% 5y	<0.0001
	KIT mut17	28	82%	78%5 y RR	26.5% 5y	
Shimada, 2006	KIT wid	38	100%	3% 4y RR	97.4% 4y	0.001
	KIT mut	8	100%	38%4y RR	50% 4y	
Qin, 2014[c]	KIT wid	89	86%	46.2% 2y CIR	65.7% 2y	0.0055
	KIT mut	57	79%	73.2% 2y CIR	48.9% 2y	
	KIT mut17	43	NR	80.6% 2y CIR	39.4%2y	0.0007
Cairoli, 2006	KIT wid	17	100%	35% 2y RR	76.5% 2y	0.017
	KIT mut	19	89%	77% 2y RR	42.1% 2y	
	KIT mut TKD	12	83%	90% 2y RR	25% 2y	
Krauth, 2014*	KIT wid	76	NR	NR	68.9% 2y	0.615
	KIT mut	19	NR	NR	71.2% 2y	

Krauth, 2014*	KIT wid	92	NR	NR	77.8% 27	
	KIT mutexon8	3	NR	NR	100% 2y	
Krauth, 2014*	KIT wid	80	NR	NR	82% 2y	0.03
	KIT mutD816	15	NR	NR	59.1% 2y	
Kim, 2013*	KIT wid and other KIT mut	73	80%	NR	80.3% 2y	0.505
	KIT mutD816	9	25%	NR	25% 2y	

[#]Data involving 78 patients with t(8,21) AML for which *c-KIT* exon 17 mutation was evaluated. *Data for sensitivity analysis.
[a]Median follow-up was 15 months for relapse rate.
[b]Data only provided for CR and OS in t(8,21)AML; correlation between exon 17 and non-exon in relapse.
[c]The related data was for adults, data for children was not provided.
NR:Not reported.
