# Supplementary material for: Prognostic Significance of KIT Mutations in Core-Binding Factor Acute Myeloid Leukemia: A Systematic Review and Meta-Analysis
Source: PLoS One. 2016 Jan 15;11(1):e0146614. doi: 10.1371/journal.pone.0146614 (PMC4714806; doi:10.1371/journal.pone.0146614)
Supplement: S4 Table — (PDF) [file pone.0146614.s009.pdf]

Outcome	AML	Studies Included, n	Participants, n	Fixed$ RRs 95%CI#	Random$ RRs 95%CI	Heterogeneity P	Heterogeneity I^2	Subgroup Differences Fixed$ P	Subgroup Differences Fixed$ I^2	Subgroup Differences Random$ P	Subgroup Differences Random$ I^2
CR											
	CBF AML	9	1131	1.24 [0.81, 1.90]	1.25 [0.75, 2.11]	0.3	16%	0.19	42.0%	0.27	19.0%
	t(8,21)	7	567	1.62 [0.90, 2.92]	1.68 [0.90, 3.15]	0.55	0%				
	inv(16)	8	564	0.91 [0.49, 1.70]	0.99 [0.50, 1.96]	0.39	4%				
	Total	9	1131	1.23 [0.80, 1.88]	1.30 [0.83, 2.06]	0.49	0%				
OS											
	CBF AML	4	610	1.15 [0.99, 1.33]	1.15 [0.99, 1.33]	0.99	0%	0.03	79.0%	0.11	60.0%
	t(8,21)	5	385	1.45 [1.12, 1.87]	1.79 [0.92, 3.49]	0.02	65%				
	inv(16)	7	547	1.04 [0.90, 1.21]	1.03 [0.85, 1.24]	0.18	33%				
	Total	7	932	1.16 [1.02, 1.32]	1.13 [0.93, 1.38]	0.03	48%				
Relapse Risk											
2y	CBF AML	7	767	1.56 [1.34, 1.82]	1.56 [1.34, 1.82]	0.81	0%	0.02	81.0%	0.25	25.0%
	t(8,21)	6	467	1.75 [1.43, 2.13]	1.75 [1.44, 2.12]	0.87	0%				
	inv(16)	5	300	1.21 [0.95, 1.54]	1.32 [0.85, 2.04]	0.11	48%				
	Total	7	767	1.50 [1.29, 1.75]	1.59 [1.33, 1.91]	0.25	20%				
5y	CBF AML	6	781	1.51 [1.26, 1.81]	1.52 [1.27, 1.82]	0.73	0%	0.04	77.0%	0.22	35.0%
	t(8,21)	5	418	1.79 [1.36, 2.36]	1.82 [1.39, 2.39]	0.79	0%				
	inv(16)	6	387	1.21 [0.95, 1.54]	1.32 [0.85, 2.04]	0.11	48%				
	Total	6	805	1.44 [1.20, 1.72]	1.56 [1.23, 1.99]	0.17	32%				

$ Abbreviations for fixed-effects model and random-effects model.

ORs for CR and RRs for OS and relapse rate.
